# Ceftriaxone attenuates hypoxic-ischemic brain injury in neonatal rats

**DOI:** 10.1186/1423-0127-18-69

**Published:** 2011-09-21

**Authors:** Pei Chun Lai, Yen Ta Huang, Chia Chen Wu, Ching-Jung Lai, Pen Jung Wang, Ted H Chiu

**Affiliations:** 1 Institute of Pharmacology and Toxicology, Tzu Chi University, Hualien, Taiwan; 2 Department of Pediatrics, Buddhist Tzu Chi General Hospital, Hualien, Taiwan; 3 Division of Surgical Critical Care Unit, Buddhist Tzu Chi General Hospital, Hualien, Taiwan; 4 Department of Research, Buddhist Tzu Chi General Hospital, Hualien, Taiwan; 5 Department of Physiology, Tzu Chi University, Hualien, Taiwan; 6 Department of Pharmacology, Tzu Chi University, Hualien, Taiwan

**Keywords:** β-lactam antibiotics, ceftriaxone, hypoxic-ischemic injury, neonatal rat, GLT1, EAAT2

## Abstract

**Background:**

Perinatal brain injury is the leading cause of subsequent neurological disability in both term and preterm baby. Glutamate excitotoxicity is one of the major factors involved in perinatal hypoxic-ischemic encephalopathy (HIE). Glutamate transporter GLT1, expressed mainly in mature astrocytes, is the major glutamate transporter in the brain. HIE induced excessive glutamate release which is not reuptaked by immature astrocytes may induce neuronal damage. Compounds, such as ceftriaxone, that enhance the expression of GLT1 may exert neuroprotective effect in HIE.

**Methods:**

We used a neonatal rat model of HIE by unilateral ligation of carotid artery and subsequent exposure to 8% oxygen for 2 hrs on postnatal day 7 (P7) rats. Neonatal rats were administered three dosages of an antibiotic, ceftriaxone, 48 hrs prior to experimental HIE. Neurobehavioral tests of treated rats were assessed. Brain sections from P14 rats were examined with Nissl and immunohistochemical stain, and TUNEL assay. GLT1 protein expression was evaluated by Western blot and immunohistochemistry.

**Results:**

Pre-treatment with 200 mg/kg ceftriaxone significantly reduced the brain injury scores and apoptotic cells in the hippocampus, restored myelination in the external capsule of P14 rats, and improved the hypoxia-ischemia induced learning and memory deficit of P23-24 rats. GLT1 expression was observed in the cortical neurons of ceftriaxone treated rats.

**Conclusion:**

These results suggest that pre-treatment of infants at risk for HIE with ceftriaxone may reduce subsequent brain injury.

## Background

Perinatal hypoxia and ischemia cause serious complications [1]. Preterm and sick infants are at high risk for brain injury and neurodevelopmental problems [2]. The hypoxia and ischemia induced brain injury in neonates is defined as hypoxic-ischemic encephalopathy (HIE) which is the leading cause of neurological sequelae in premature infants. The pathophysiology of HIE includes energy failure, intracellular calcium accumulation, glutamate and nitric oxide neurotoxicity, lipid peroxidation, free radical formation, and inflammation [3,4]. As the survival rate of premature infants increased since 1990s, increased risk of significant neurodevelopmental impairment was also noted [5]. Intervention strategies to HIE include hypothermia and erythropoietin therapy, which reduce neurological damage in animal models of HIE [3]. In recent human studies, therapeutic hypothermia demonstrated a significant reduction of the risk of death and neurological impairment at 18 months of age [6]. But, there was no significant difference in the severe neurodevelopmental delay in the survivors. Further studies are warranted to improve the neurological sequelae after HIE damage.

Five subtypes of glutamate transporter (excitatory amino acid transporters; EAAT 1-5) have been characterized in human. In other mammalian species, GLAST, GLT1, and EAAC1 have been found to correspond to human EAAT1, 2, and 3, respectively [7]. The glutamate transporters are responsible for the rapid removal of glutamate from the extracellular space [8]. GLT1 (or EAAT2), expressed mainly in the glial cells, plays a principal role in removing the excessive glutamate from the extracellular space [9,10]. Some pathological conditions have been associated with alteration in EAAT2 expression, such as amyotrophic lateral sclerosis [11], Alzheimer's disease [12], and Huntington disease [13]. Interventions targeting on the glutamate transporter have been conducted [14,15]. Several antibiotics were found to upregulate significantly GLT1 expression. Ceftriaxone, a third generation cephalosporin, was one of the antibiotics found to exert neuroprotection by increasing GLT1 expression in an animal model of amyotrophic lateral sclerosis [14]. Ceftriaxone also exhibited beneficial effects in *in vitro *and *in vivo *model of stroke [16,17]. However, there is no report investigating the effects of ceftriaxone in neonatal HIE.

In this study, we used a rodent model of neonatal HIE with unilateral carotid artery ligation and subsequent exposure to 8% oxygen for 2 hrs on postnatal day 7 rats (the day of birth was designated as P0). The P7 neonatal rat is comparable to the 34 weeks old human fetus [18]. Different dosages of ceftriaxone were used in these rat pups to clarify if ceftriaxone treatment could offer neuroprotection against the hypoxic-ischemic brain injury. Our results indicate that pretreatment with ceftriaxone in neonatal rats can reverse hypoxic-ischemic induced morphological and functional alterations.

## Methods

### Animals

This study was approved by the Institutional Animal Care and Use Committee of Tzu Chi University. Pregnant Sprague-Dawley (SD) rats were housed in individual cages with 12 hrs light/dark cycle at 22 ± 2°C with free access to food and water. After normal delivery, the size of the litter was adjusted to 10 male rat pups to eliminate the gender difference of neonatal HIE [19].

### Neonatal rat model of hypoxic-ischemic encephalopathy and treatment design

The neonatal rat model of HIE as described previously [20] was followed with minor modifications. Briefly, a less than 1 cm longitudinal midline incision of the neck was performed under ether anesthesia on P7 rats. The left carotid artery was exposed and permanently ligated with 4-0 surgical silk. The surgery lasted less than 5 min. Animals with excessive bleeding were excluded. The rat pups were returned to home cage with their dam for 1 hr followed by exposure to hypoxia (92% N_2 _+ 8% O_2_) for 2 hrs by placing them in an airtight chamber partially submersed in a 37°C water bath. At the end of 2 hrs hypoxia, the pups were returned to their dam again for recovery.

Ceftriaxone (Sigma Chemical Co, St. Louis, MO) was dissolved in sterile water and dosage of 50, 100 or 200 mg/kg was given intraperitoneally to three different groups of randomly assigned rats. Rats were pre-treated daily with ceftriaxone for 2 days followed by a third dose given 1 hr before ligation and hypoxia. These animals were assigned to the drug treatment group. Animals in the control or normal group were treated with the same volume of saline. Similar to previous report [20], the control animals received sham operation that consisted of left carotid artery exposure without ligation and then exposed to hypoxia for 2 hrs.

### Brain tissue preparation

Rats were administered intraperitoneally an overdose of 10% chloral hydrate on P14, and perfused transcardially with 20 ml ice-cold saline followed by 20 ml 4% paraformaldehyde in 0.1 M phosphate buffer (pH 7.4). Brains were removed and fixed in 4% paraformaldehyde in 0.1 M phosphate buffer overnight at 4°, transferred sequentially to 15% sucrose and then 30% sucrose in 0.1 M phosphate buffer until the brains sank for cryoprotection. Brains were then embedded in O.C.T (Sakura, Torrance, CA) and stored at -80°C for immunohistochemistry and immunofluorescence studies. The brains were sectioned coronally into 10 μm slices with a cryostat (Leica CM3050, Leica Instruments, Nussloch, Germany) at -20°C to -22°C. Brain sections were mounted onto superfrost plus slides (Menzel Gläser, Braunschweig, Germany) and stored at -20°C until use.

### Nissl stain and brain injury score

Coronal brain sections corresponding to plate 18 and 31 according to the rat brain atlas [21] were examined. The selected brain sections were stained with 0.5% cresyl violet acetate (Sigma, #C1791). We used a standard histological scoring system for evaluating the rodent model of HIE [22]. Brain sections were scored according to: 0 = no detectable lesion, 1 = small focal area of neuronal cell loss, 2 = columnar damage in the cortex involving the layers II-IV or moderate neuronal cell loss, and 3 = cystic infarction and gliosis. Eight brain regions (hippocampus: CA1, CA2, CA3, dentate gyrus; anterior and middle regions of cortex; striatum and thalamus) were evaluated, scored, and the scores summed to yield the final scores, ranging from 0 to 24 for each animal.

### Immunohistochemical staining

Conventional procedures were followed with some modifications. Briefly, brain sections were rehydrated with decreasing ethanol concentrations (100%, 95%, 75%, 50%) for 5 min each and washed with phosphate-buffered saline (PBS). Background staining was blocked using protein block (NovoLink™ Polymer Detection System, Novocastra, Newcastle Upon Tyne, UK). After washing with PBS, sections were incubated with primary antibodies with the following dilution ratio: anti-MBP (1:200, sc-13914, Santa Cruz Biotechnology Inc., Santa Cruz, CA), and anti-EAAT 2 (1:100, #3838s, Cell Signaling Technology, Danvers, MA). Sections were treated for 2 hrs at room temperature with horseradish peroxidase-conjugated secondary antibodies (1:1000, sc-2352, Santa Cruz) for MBP (myelin basic protein), or incubated with NovoLink™ Polymer for 30 min for EAAT2. Substrate 3, 3'-diaminobenzidine (DAB, Dako, Denmark) was added for less than 5 min. Slides were examined with a computer-assisted Olympus BX51 microscope and images were taken with an Olympus DP72 microscope digital camera.

### Neurobehavioral tests

#### Cliff avoidance test

Cliff avoidance test was performed on P14 rats for assessing the integrity of exteroceptive input and locomotor output [23]. Rats were placed in the edge of a platform (30 cm × 30 cm × 30 cm) with forepaws and chest extending over the edge. The latency of the rats to turn away or withdraw from the edge was recorded. If the pups fell from the platform or did not response within 60 seconds, the latency was recorded as 60 seconds.

#### Negative geotaxis test

Negative geotaxis test examines the sensorimotor function of neonatal rats [24]. The P14 rat pups were placed on a 30-degree inclined plate with rough surface. Their heads were facing downward. The latency to turned 180 degree to an upward direction was recorded. The maximum duration of recording was 90 seconds.

#### Rotarod performance test

The rotarod test was used for evaluating the motor and coordination performance in animals [24]. The test was performed on P21 rats with the rolling rate of 5 rpm. Rats were placed on the rod and observed for 3 min. The duration of rats holding on the rod without falling down was recorded as the day one trial. On the following day P22, rats were placed on the rod again with the rolling rate of 5 rpm. The duration of holding on the rod was recorded.

#### Step-down passive avoidance test

Step-down passive avoidance test was used to measure the learning and memories in animals [23]. Rats were place in a 30 cm × 30 cm × 30 cm black acrylic chamber. The floor was made of paralleled 2 mm in diameter and 1 cm apart from each other stainless steel rods. The floor of steel rods was connected to an electric shock generator. At the center of the floor, an acrylic board (15 cm × 15 cm × 2.5 cm) was placed and served as a safe platform on the floor. In session one, each animal (P23) was placed initially on the safe acrylic board. When rats stepped down to the metal rods, they received an electrical foot shock (1sec, 0.5 mA). Rats stepped down and up on the safe board, and the latency of stepping down till the rats stayed on the board for 2 min were recorded. Session two (P24) was conducted one day later. Rats were placed on the safe board and the latency of each animal stayed on the safe board before starting to step down to the metal rods was recorded as retention time. If the animal stayed on the safe board without stepping down to the metal rods, the latency is recorded as 2 min. Following the latency of staying on safe board, the duration of stepping down till the animal again stayed on the board for 2 min was recorded. If the animal stayed still on the safe board after placing on the safe board for more than 5 min, the duration of stepping down was recorded as zero.

### Western blot

Conventional methodologies were used. Particulate fractions from P7 brain homogenates were solubilized with protein extraction solution (PRO-PREP™ protein extraction solution, iNtRON Biotechnology Inc., Seoul, Korea). After 30 min incubation, the sample was centrifuged at 13,000 rpm (Allegra™ 21R centrifuge, Beckman Coulter, Palo Alto, CA) at 4°C for 10 min. The supernatant consisted of the solubilized membrane portion of tissue.

Primary antibody, anti-EAAT2 (1:1000, #3838s, Cell Signaling Technology Inc., Danvers, MA), was used. Expression of α-tubulin (1:2000, sc-8035, Santa Cruz) was used as internal standard. Immunocomplexes were observed with enhanced chemiluminescent detection.

### TUNEL assay

P14 post HIE rat brain tissue was evaluated with in situ apoptosis detection kit (NeuroTACS™ II; R&D Systems, Minneapolis, MN) as recommended by the manufacturer. Brain sections corresponding to plates 31 of the rat brain atlas [21] were chosen for evaluating the hippocampal neuronal apoptosis. Hippocampus (CA1, CA2 and CA3) ipsilateral to carotid artery ligation was examined and the number of apoptotic cells was calculated under 200X light microscope. TUNEL positive cells were counted in 3 separate fields of CA1, CA2 and CA3 areas and summated for each animal.

### Image analysis and statistical analysis

Image J of NIH was used for densitometric analysis of Western blots and MBP expression density in the external capsule between ipsi- and contra-lateral sides to the carotid ligation. All data were expressed as mean ± standard error of mean (SEM). Statistical comparison between groups was carried out using one way ANOVA or Student's t test. A *p *value of less than 0.05 was considered statistically significant.

## Results

### Ceftriaxone protected against hypoxic-ischemic brain injury in neonatal rats

**Figure 1:**
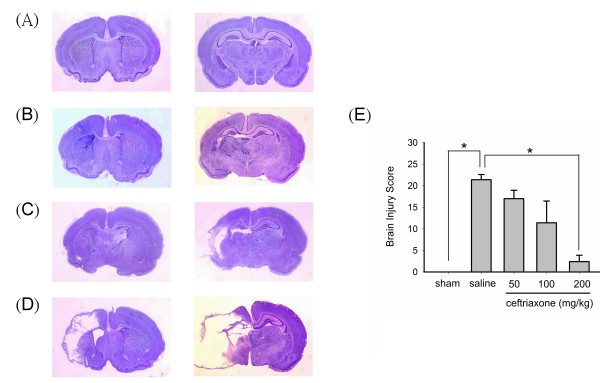
**Ceftriaxone protected against hypoxic-ischemic brain injury in neonatal rats**. *A-D*, Nissl stains of coronal brain sections from rat sacrificed on P14 after left carotid artery ligation and subsequent two-hour hypoxia on P7. Panel A, B, C and D represents two coronal brain sections illustrating the brain injury score of 0, 1, 2, and 3, respectively. *E*, Pre-treatment with ceftriaxone dose-dependently reduced brain injury scores and ceftriaxone with dosage 200 mg/kg significantly reduced the hypoxic-ischemic brain injury. one-way ANOVA, p = 0.0016 (n = 5 in each group); *: *p *< 0.05. Sham denotes animals received left carotid artery exposure without ligation and followed by 2 hrs of hypoxic challenge. Saline refers to hypoxic-ischemic animals received saline injection.

Figure 1 shows the Nissl staining of coronal brain sections from P14 rat after left carotid artery ligation and subsequent exposure to 8% oxygen for 2 hrs on P7. Panels A to D show representative brain injury score increasing from 0 to 3. Brain injury score was significantly and dose-dependently attenuated by pre-treatment with 3 different dosages of ceftriaxone 48 hrs prior to hypoxia-ischemia challenge (Figure 1E). Ceftriaxone at 200 mg/kg almost completely reversed the hypoxia-ischemia induced brain damage.

### Ceftriaxone attenuated hypoxic-ischemic white matter injury in neonatal rats

**Figure 2:**
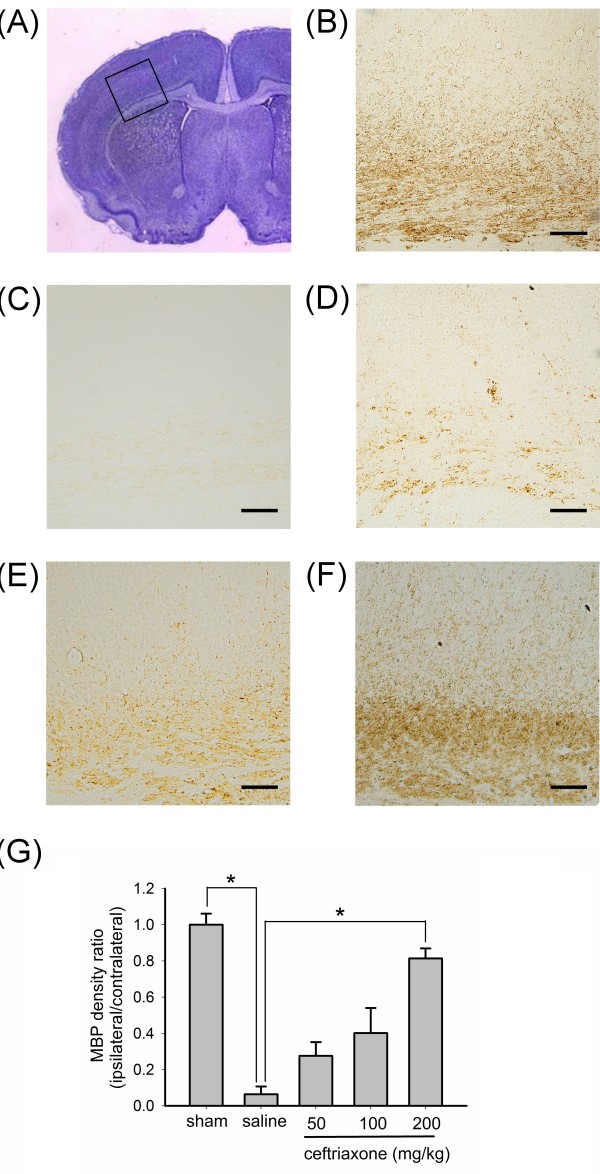
**Ceftriaxone attenuated hypoxic-ischemic white matter injury in neonatal rats**. A, the rectangular inset indicated the area of analysis. B-F, immunohistochemical staining of myelin basic protein (MBP) in external capsule of left coronal brain sections from P14 rats. (B: sham operation, C: saline. D: ceftriaxone 50 mg/kg, E: ceftriaxone 100 mg/kg, F: ceftriaxone 200 mg/kg). scale bar = 100 μm. G, Ratio of MBP density (ipsilateral/contralateral) showed significant reduction in saline treated group. Ceftriaxone treatment dose-dependently attenuated the MBP loss and pre-treatment with ceftriaxone 200 mg/kg showed statistically significant rescue of MBP loss compared to saline group. one-way ANOVA, p = 0.0001 (n = 5 in each group); *: *p *< 0.05. The definitions for sham and saline groups are the same as those in Figure 1.

White matter damage was also observed in this rodent model of hypoxic-ischemic brain injury. The white matter injuries included delayed pre-oliogodendrocytes maturation, loss of MBP, white matter cell death, and gliosis [25]. Figure 2 shows the result of MBP immunostaining from P14 rat brain. The inset in panel A shows the Nissl stain of external capsule region examined for MBP staining following ipsilateral ligation, and the enlarged photographs of MBP staining were shown from panel B to F. Large extent of MBP loss was observed in the P14 rat brain ipsilateral to the carotid ligation (panel C). Pre-treatment with ceftriaxone attenuated the MBP loss of P14 rats in a dose-dependent manner (panel D-F) with the highest ceftriaxone dose (200 mg/kg) almost completely rescued the white matter injury (panel F vs. panel B). The relative density of MBP in the ischemic-hypoxic side was calculated as the ratio of the MBP staining level in the ipsilateral side divided by that of the contralateral side of the same tissue section. Figure 2G shows quantitatively that pre-treatment with ceftriaxone significantly attenuated the MBP loss in P14 rats.

### Ceftriaxone reduced hypoxic-ischemic cell damage in the hippocampus

**Figure 3:**
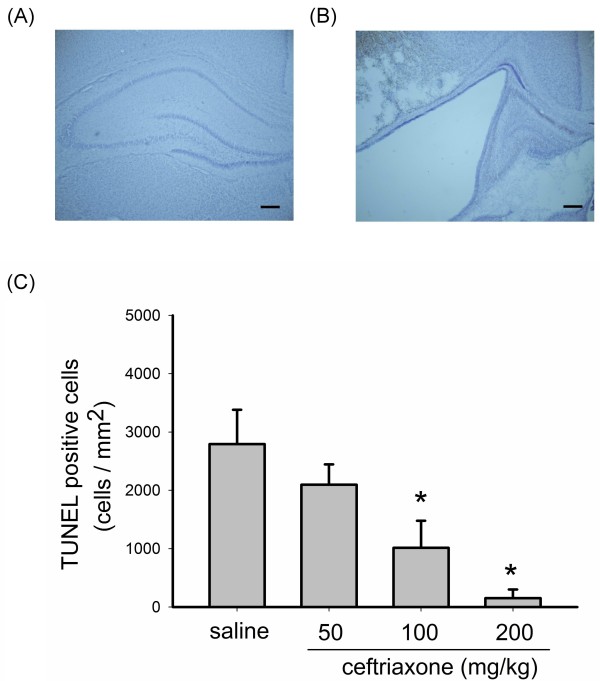
**Ceftriaxone reduced hypoxic-ischemic cell damage in the hippocampus**. A, B, in situ cell death detection by TUNEL reaction in hippocampus of P14 rat brains counter-stained with hematoxylin. (A: ceftriaxone 200 mg/kg, B: saline, scale bar = 200 μm). C, the brains were evaluated under 200X light microscope in 3 separate fields each of CA1, CA2, and CA3 (total 9 fields) and summed for each animal. Pre-treatment with 100 mg/kg and 200 mg/kg ceftriaxone significantly reduced the TUNEL positive cell. one-way ANOVA, p = 0.0017 (n = 5 in each group); *: *p *< 0.05. Saline group refers to animals received hypoxic-ischemic procedures and saline administration.

TUNEL assay was performed in coronal brain slices of P14 rats. Hippocampal cell loss was noted after HIE (Figure 3B). The HIE induced hippocampal cell damage included both necrosis and apoptotic cell damage [26]. TUNEL assay was evaluated under 200X light microscope in 3 fields each of hippocampal CA1, CA2 and CA3 area, which were summed for each animal. Figure 3C demonstrates that pre-treatment with ceftriaxone reduced the TUNEL positive cells in hippocampal area in a dose-dependent manner with statistical significance found for 100 and 200 mg/kg dosages.

### Ceftriaxone improved learning and memory performance in rats exposed to HIE

**Figure 4:**
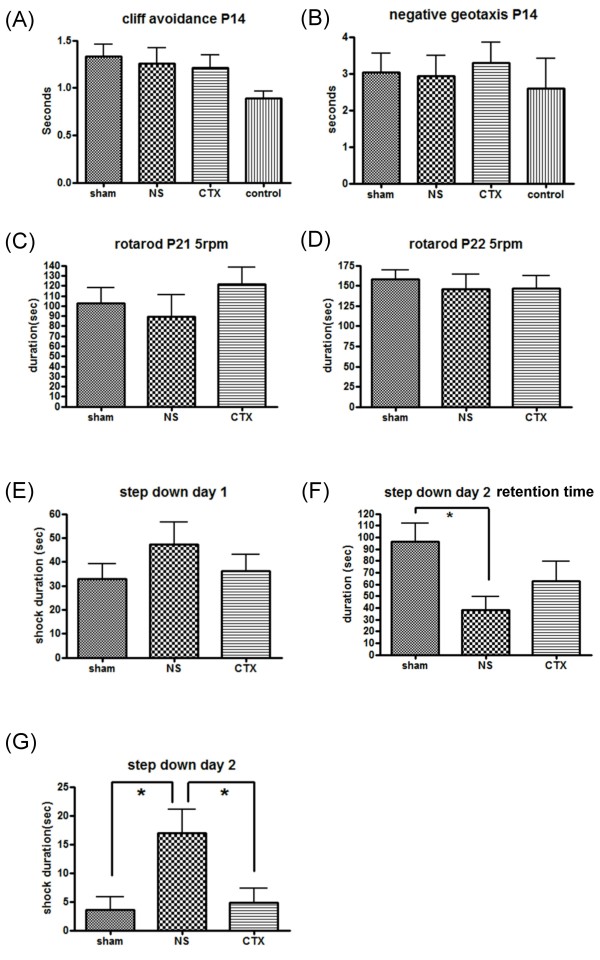
**Ceftriaxone improved performance of step-down passive avoidance test**. A: cliff avoidance test, B: negative geotaxis test, C: rotarod test with 5 rpm on P21, D: rotarod test with 5 rpm on P22, E: step-down passive avoidance test session one on P23, F, G: step-down passive avoidance test session two on P24. F: Significant reduction of retention time was observed in saline group compared to sham group. Ceftriaxone treatment improved the duration of rats stayed on safe board in step-down passive avoidance test without statistical significance. G: Ceftriaxone significantly reduced the foot shock duration after HIE injury. (Abbreviation: NS: saline group, animals received hypoxic-ischemic procedures and given saline injection, CTX: ceftriaxone 200 mg/kg group, Sham: sham operated group) *: p < 0.05, Student's t test. (n = 10 in each group)

Based on the above morphological observations that pre-treatment with 3 dosages of ceftriaxone reversed the brain damage caused by ischemic-hypoxic insult, this treatment protocol was followed to evaluate its effects on several behavioral tests reflecting motor, learning, and memory functions. Figure 4 shows that ceftriaxone was without effect on cliff avoidance on P14 (Figure 4A), negative geotaxis on P14 (Figure 4B), rotarod test on P21 and P22 (Figure 4C and 4D) or the first session of step-down passive avoidance on P23 rats (Figure 4E). However, in session two trial of step-down passive avoidance (P24 rats), pre-treatment with ceftriaxone significantly reduced the duration of foot shock (Figure 4G).

### Ceftriaxone did not alter GLT1 protein expression in rat brain homogenate

**Figure 5:**
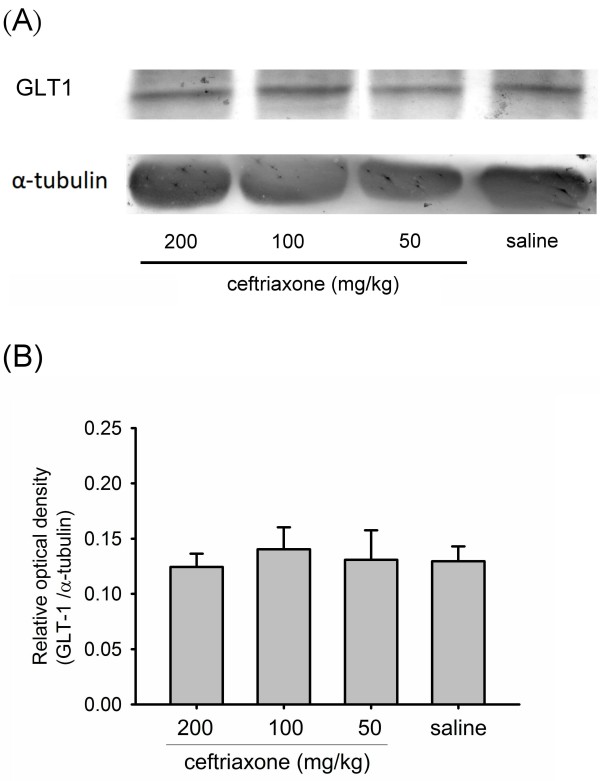
**Pre-treatment with ceftriaxone did not increase GLT1 protein expression in neonatal rat brain tissue**. *A*, GLT1 protein expression in P7 rat brain tissue following pre-treatment with different dosages of ceftriaxone and saline; *B*, statistic analysis showed no difference in GLT1 protein expression among these groups. one-way ANOVA, p = 0.95 (n = 5 in each group). Saline group, animals received hypoxic-ischemic procedures and given saline injection.

After pre-treatment with different dosages (50, 100 or 200 mg/kg) of ceftriaxone or saline, the membrane portion of P7 rat brain lysate was used for measuring the expression of GLT1 protein. A representative immunoblotting is demonstrated in Figure 5A. The expression of GLT1 was not altered by pre-treatment with ceftriaxone (Figure 5B).

### Ceftriaxone induced the expression of GLT1 in the cortical neurons of neonatal rat brain

**Figure 6:**
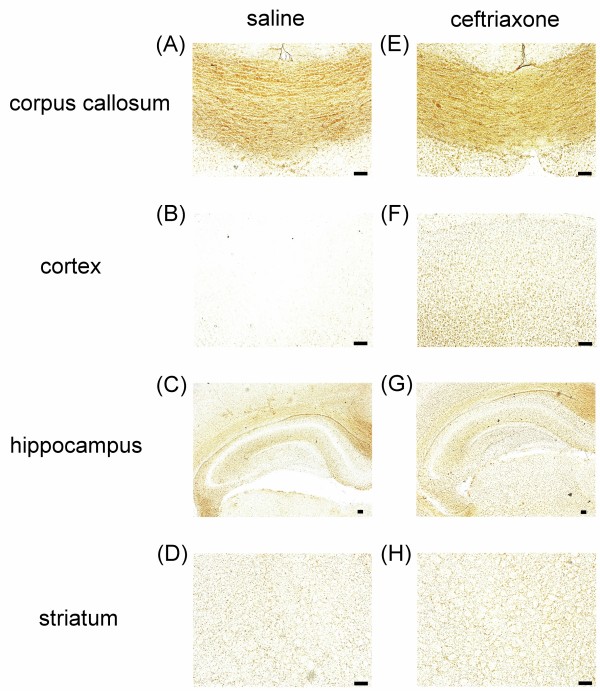
**Regional difference of GLT1 protein in neonatal rats among ceftriaxone treated and saline groups**. Immunohistochemical staining of GLT1 in saline and ceftriaxone treated groups in P7 rat brain. (ceftriaxone: pre-treatment with 3 dosages of 200 mg/kg ceftriaxone, saline: pre-treatment with 3 dosages of saline). A, E: corpus callosum. B, F: cerebral cortex. C, G: hippocampus. D, H: striatum. Increased GLT1 protein expression in cerebral cortex was noted in ceftriaxone group compared to saline group. There was no significant difference in GLT1 expression in corpus callosum, hippocampus and striatum. Scale bar = 100 μm.

**Figure 7:**
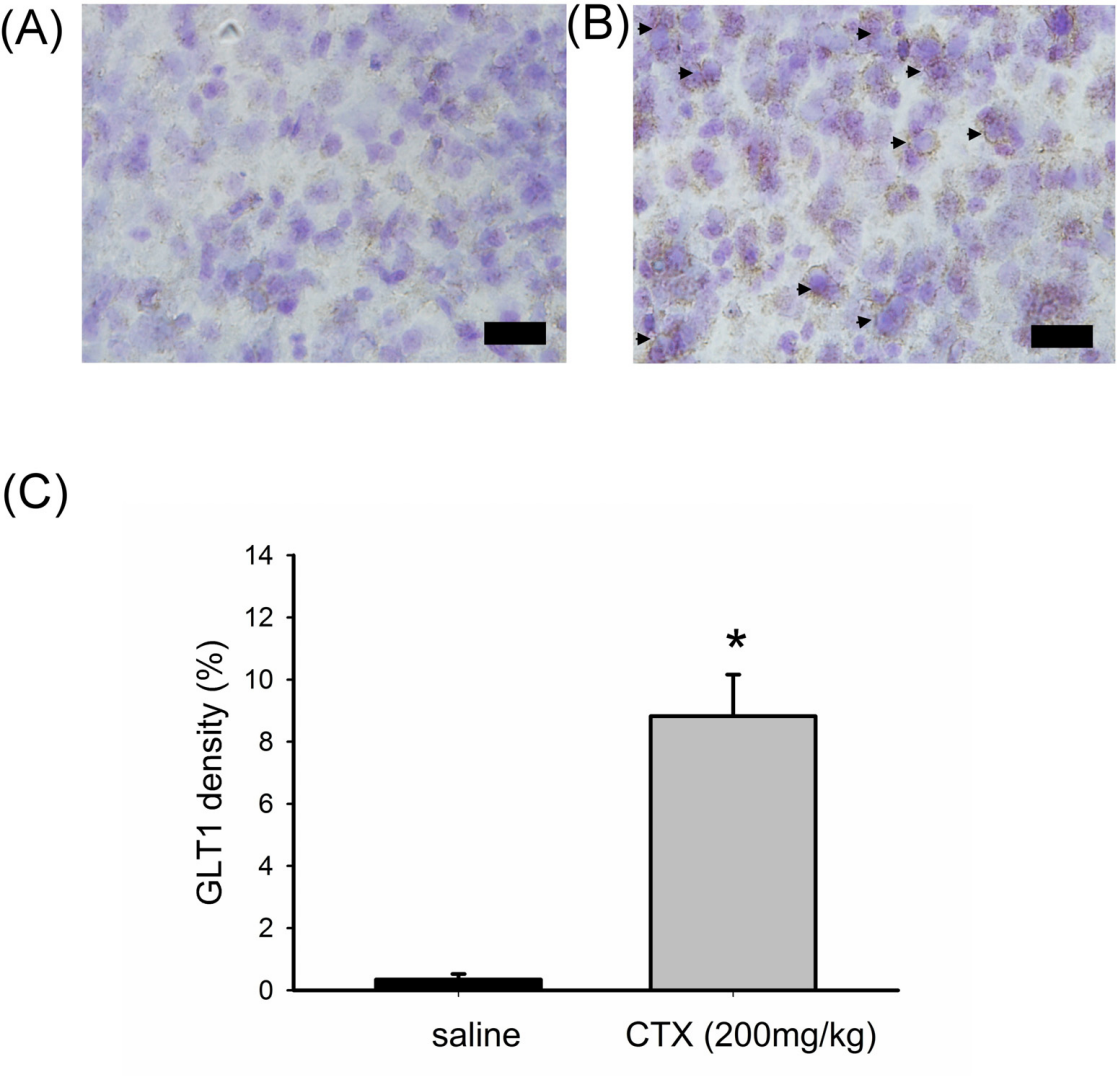
**Pre-treatment with ceftriaxone induced the GLT1 protein expression in cerebral cortical neuron**. Immunohistochemistry staining of GLT1 (DAB: brown) and counter-staining with Nissl stain (blue) in cerebral cortex of P7 rat. A: pre-treatment with 3 dosages of saline, B: pre-treatment with 3 dosages of 200 mg/kg ceftriaxone. Arrow indicated neuronal expression of GLT1 protein. Scale bar = 20 μm. C: percentage of area of immunohistochemical staining for GLT1 under 400X light microscope in saline and ceftriaxone treated groups. (n = 3 in saline and n = 4 in ceftriaxone group, *: *p *< 0.05.) Saline group, animals received hypoxic-ischemic procedures and given saline injection

We further examined if there was regional difference in the expression of GLT1 protein that could explain at least partly the neuroprotection mediated by ceftriaxone administration. Pre-treatment with 3 dosages of 200 mg/kg ceftriaxone was followed since it significantly reduced the histological and behavioral deficits. Immunohistochemial study with anti-EAAT2 antibody was carried out in brain slides to reveal the regional difference of GLT1 expression between ceftriaxone treated and saline group. Figure 6 demonstrates immunohistochemical staining of GLT1 in saline and ceftriaxone treatment groups. Each panel showed different regions of brain section (A,E: corpus callosum; B,F: cerebral cortex; C,G: hippocampus and D,H: striatum). Figure 6B shows that cerebral cortex from control P7 brain expressed little GLT1 protein. Figure 6F demonstrates that ceftriaxone pre-treatment, however, induced GLT1 protein expression in this area. After counterstained with Nissl stain, the GLT1 protein was found to be expressed in cortical neuronal cells (Figure 7B arrow). Image J was used to analyze the percentage of EAAT2 (GLT1) immunoreactive area of P7 rat cortex under 400X light microscope in saline and ceftriaxone pre-treated groups. Ceftriaxone pre-treatment significantly induced GLT1 protein expression in cortical neuron (Figure 7, P = 0.031).

## Discussion

In this study, we showed that neonatal ischemic-hypoxic brain damage can be attenuated by pre-treatment with ceftriaxone. Our data are consistent with similar approaches reported in the literature [27]. However, the present study is the first to investigate the utility of ceftriaxone in a neonatal rat model of ischemic-hypoxic brain damage. Since ceftriaxone is a FDA approved drug and exhibits relatively few adverse effects, the potential clinical benefit of ceftriaxone and related antibiotics in human neonatal HIE warrants further investigation.

For the pathophysiology of neonatal HIE, glutamate neurotoxicity remains an important issue in subsequent calcium influx, free radical formation, necrosis, and apoptosis [28]. During brain development, glutamate plays an important role in oligodendrocyte maturation and myelination, but can lead to detrimental consequences from excessive release after HIE [29,30]. The blockade of glutamate receptor by antagonists improved white matter injury [25,31,32]. Experimental drugs that block NMDA-type glutamate receptor could protect the brain from severe hypoxic-ischemic insults if given before or shortly after the insult, but were ineffective if administration was delayed for more than several hrs [33-36]. These data suggest that downstream events quickly become self-sustaining after neonatal HIE [28].

An alternative approach to reduce glutamate neurotoxicity is to augment the glutamate reuptake. GLT1 glutamate transporter plays a major role in the reuptake of extracellular glutamate and is expressed mainly in mature astrocytes although minor expression has been found in neurons, microglias, and oligodendrocytes. But, astrocytes in immature human or rat brain do not express EAAT2 or GLT1 [37-40]. GLT1 expression is very low in the early postnatal period and reaches adult levels in hippocampus at 3-4 weeks old in rat brain tissue and hippocampus [16,40,41]. The roles of GLT1 in immature brain remained unclear. In human premature infant, expression of EAAT2 was observed in pre-oligodendrocytes which might be the cause of white matter vulnerability to HIE injury. Upregulation of EAAT2 (or GLT1) was observed in reactive astrocytes and macrophages in the area of periventricular leukomalacia (PVL) [38,39]. In a rat model of neonatal HIE, altered expression of glutamate transporter and decreased GLT1 expression were observed in the area of ischemic core [42]. Prolonged hypoxia reduced GLT1 expression in astrocytes resulting in the accumulation of extracellular glutamate [43]. Furthermore, functional reversal of glutamate transporter in glial cells occurred during hypoxia and ischemia also contributed to the excessive extracellular glutamate toxicity [44]. In this study, we used a FDA approved beta-lactam antibiotic, ceftriaxone. It has been found that a 5-7 days course of ceftriaxone increased GLT1 protein expression in organotypic spinal cord slice cultures, neuronal culture under glucose-oxygen deprivation, human fetal astrocytes culture, and in the rat brain [14]. These results have been confirmed in hippocampal slice culture and in rat brains [27,45]. In contrast, upregulation of GLT1 expression by ceftriaxone treatment was not observed in a rat stroke model, in organotypic hippocampal slices or in a mouse model of multiple sclerosis [16,17,46]. Ceftriaxone may offer neuroprotection via other mechanisms, such as increased GLT1 transporter activity, stimulation of neurotrophin release or reduction of T cell activation by modulation of cellular antigen-presentation [17,46].

In our studies, GLT1 protein expression in the whole brain lysate of P7 rat did not change after ceftriaxone treatment. But, immunohistochemical study showed that pre-treatment with ceftriaxone induced GLT1 protein expression in cerebral cortex of P7 rat. GLT1 expressed in neurons of the brain is observed during early stages of development and is present during axonal growth, which disappears on maturation [47]. The role of GLT1 in immature neuron remains to be investigated. In mature rat brain, neuronal expression of GLT1 protein and mRNA had also been found and might play a role in the pathophysiology of excitotoxicity [48-50]. But, in our study, pretreatment with ceftriaxone increased expression of GLT1 in the cerebral cortical neuron of P7 rat. Neuronal expression of GLT1 protein was confirmed after counterstained with Nissl stain. The presence of GLT1 in neurons might enhance glutamate uptake after hypoxic-ischemic injury. However, other mechanisms, such as enhanced GLT activity and/or anti-inflammatory effect of ceftriaxone, cannot be excluded.

Several behavioral paradigms mimic the childhood behavior in human were examined in the young rat. No difference was detected in the primitive reflexes (cliff avoidance and negative geotaxis test) and motor function test among treatment, vehicle, and sham groups. On the other hand, significant improvement in step-down passive avoidance test was found after ceftriaxone treatment. The difference of behavior between HIE group and normal control group included long-lasting sensorimotor and locomotor deficits [51]. But, unlike human, rats exposed to HIE injury did not exhibit gross motor function deficit in some studies although some permanent deficit has also been observed [24]. This may be due to a higher degree of plasticity of neonatal rat brain compared with that of human brain. Step-down passive avoidance reflects learning and memory function. In our studies, ceftriaxone rescued hippocampal cells from apoptosis which may contribute to improved step-down passive avoidance results.

Pre-treatment with agents prior to the appearance of pathological changes remains debatable in clinical application. But, in premature baby, pre-treatment may be acceptable because pregnant mother usually receives tocolysis for prevention of preterm birth. In addition, ceftriaxone exhibits antibiotic effect which could eliminate the pathogens if maternal chorioamnionitis is diagnosed [52] since ceftriaxone effectively crosses the placenta [53].

## Conclusions

In conclusion, pre-treatment with ceftriaxone for 48 hrs prior to hypoxic-ischemic brain injury in neonatal rats reduced brain injury score, improved myelination, decreased hippocampal apoptotic cell death, and restored learning and memory deficit. Induction of GLT1 protein expression in cerebral cortex after ceftriaxone pre-treatment was observed in P7 rats, which might partially explain the neuroprotective effect of ceftriaxone. Ceftriaxone may be an effective therapeutic agent for the treatment of neonatal HIE.

## Competing interests

The authors declare that they have no competing interests.

## Authors' contributions

PCL and YTH carried out animal study, participated in the immunohistochemistry, performed the statistical analysis, and drafted the manuscript. CCW carried out the West blot. PJW and THC conceived the study, participated in its design and coordination, and helped to draft the manuscript. All authors read and approved the final manuscript.

## References

[B1] van Bel F Groenendaal F Long-term pharmacologic neuroprotection after birth asphyxia: where do we stand? Neonatology 2008 94 203 210 10.1159/000143723 18832856

[B2] van Handel M Swaab H de Vries LS Jongmans MJ Long-term cognitive and behavioral consequences of neonatal encephalopathy following perinatal asphyxia: a review Eur J Pediatr 2007 166 645 654 10.1007/s00431-007-0437-8 17426984 PMC1914268

[B3] Perlman JM Intervention strategies for neonatal hypoxic-ischemic cerebral injury Clin Ther 2006 28 1353 1365 10.1016/j.clinthera.2006.09.005 17062309

[B4] Vannucci RC Connor JR Mauger DT Palmer C Smith MB Towfighi J Vannucci SJ Rat model of perinatal hypoxic-ischemic brain damage J Neurosci Res 1999 55 158 163 10.1002/(SICI)1097-4547(19990115)55:2<158::AID-JNR3>3.0.CO;2-1 9972818

[B5] Wilson-Costello D Friedman H Minich N Fanaroff AA Hack M Improved survival rates with increased neurodevelopmental disability for extremely low birth weight infants in the 1990s Pediatrics 2005 115 997 1003 10.1542/peds.2004-0221 15805376

[B6] Edwards AD Brocklehurst P Gunn AJ Halliday H Juszczak E Levene M Strohm B Thoresen M Whitelaw A Azzopardi D Neurological outcomes at 18 months of age after moderate hypothermia for perinatal hypoxic ischaemic encephalopathy: synthesis and meta-analysis of trial data BMJ 2010 340 c363 369 10.1136/bmj.c363 20144981 PMC2819259

[B7] Arriza JL Fairman WA Wadiche JI Murdoch GH Kavanaugh MP Amara SG Functional comparisons of three glutamate transporter subtypes cloned from human motor cortex J Neurosci 1994 14 5559 5569 7521911 10.1523/JNEUROSCI.14-09-05559.1994 PMC6577102

[B8] Shigeri Y Seal RP Shimamoto K Molecular pharmacology of glutamate transporters, EAATs and VGLUTs Brain Res Brain Res Rev 2004 45 250 265 15210307 10.1016/j.brainresrev.2004.04.004

[B9] Rao VL Dogan A Todd KG Bowen KK Kim BT Rothstein JD Dempsey RJ Antisense knockdown of the glial glutamate transporter GLT-1, but not the neuronal glutamate transporter EAAC1, exacerbates transient focal cerebral ischemia-induced neuronal damage in rat brain J Neurosci 2001 1 1876 1883 10.1523/JNEUROSCI.21-06-01876.2001 PMC6762628 11245672

[B10] Rothstein JD Dykes-Hoberg M Pardo CA Bristol LA Jin L Kuncl RW Kanai Y Hediger MA Wang Y Schielke JP Welty DF Knockout of glutamate transporters reveals a major role for astroglial transport in excitotoxicity and clearance of glutamate Neuron 1996 16 675 686 10.1016/S0896-6273(00)80086-0 8785064

[B11] Rothstein JD Van Kammen M Levey AI Martin LJ Kuncl RW Selective loss of glial glutamate transporter GLT-1 in amyotrophic lateral sclerosis Ann Neurol 1995 38 73 84 10.1002/ana.410380114 7611729

[B12] Li S Mallory M Alford M Tanaka S Masliah E Glutamate transporter alterations in Alzheimer disease are possibly associated with abnormal APP expression J Neuropathol Exp Neurol 1997 56 901 911 10.1097/00005072-199708000-00008 9258260

[B13] Arzberger T Krampfl K Leimgruber S Weindl A Changes of NMDA receptor subunit (NR1, NR2B) and glutamate transporter (GLT1) mRNA expression in Huntington's disease--an in situ hybridization study J Neuropathol Exp Neurol 1997 56 440 454 10.1097/00005072-199704000-00013 9100675

[B14] Rothstein JD Patel S Regan MR Haenggeli C Huang YH Bergles DE Jin L Dykes Hoberg M Vidensky S Chung DS Toan SV Bruijn LI Su ZZ Gupta P Fisher PB Beta-lactam antibiotics offer neuroprotection by increasing glutamate transporter expression Nature 2005 433 73 77 10.1038/nature03180 15635412

[B15] Sheldon AL Robinson MB The role of glutamate transporters in neurodegenerative diseases and potential opportunities for intervention Neurochem Int 2007 51 333 355 10.1016/j.neuint.2007.03.012 17517448 PMC2075474

[B16] Lipski J Wan CK Bai JZ Pi R Li D Donnelly D Neuroprotective potential of ceftriaxone in in vitro models of stroke Neuroscience 2007 146 617 629 10.1016/j.neuroscience.2007.02.003 17363173

[B17] Thone-Reineke C Neumann C Namsolleck P Schmerbach K Krikov M Schefe JH Lucht K Hortnagl H Godes M Muller S Rumschussel K Funke-Kaiser H Villringer A Steckelings UM Unger T The beta-lactam antibiotic, ceftriaxone, dramatically improves survival, increases glutamate uptake and induces neurotrophins in stroke J Hypertens 2008 26 2426 2435 10.1097/HJH.0b013e328313e403 19008722

[B18] Hagberg H Bona E Gilland E Puka-Sundvall M Hypoxia-ischaemia model in the 7-day-old rat: possibilities and shortcomings Acta Paediatr 1997 422 Suppl 85 88 10.1111/j.1651-2227.1997.tb18353.x 9298801

[B19] Nunez J Yang Z Jiang Y Grandys T Mark I Levison SW 17beta-estradiol protects the neonatal brain from hypoxia-ischemia Exp Neurol 2007 208 269 276 10.1016/j.expneurol.2007.08.020 17950281 PMC2194656

[B20] Rice JE Vannucci RC Brierley JB The influence of immaturity on hypoxic-ischemic brain damage in the rat Ann Neurol 1981 9 131 141 10.1002/ana.410090206 7235629

[B21] George Paxinos CW The rat brain in stereotaxis of coordinates 1986 Orlando, Florida, Academic press

[B22] Sheldon RA Sedik C Ferriero DM Strain-related brain injury in neonatal mice subjected to hypoxia-ischemia Brain Res 1998 810 114 122 10.1016/S0006-8993(98)00892-0 9813271

[B23] Fan LW Lin S Pang Y Lei M Zhang F Rhodes PG Cai Z Hypoxia-ischemia induced neurological dysfunction and brain injury in the neonatal rat Behav Brain Res 2005 165 80 90 10.1016/j.bbr.2005.06.033 16140403

[B24] Lubics A Reglodi D Tamas A Kiss P Szalai M Szalontay L Lengvari I Neurological reflexes and early motor behavior in rats subjected to neonatal hypoxic-ischemic injury Behav Brain Res 2005 157 157 165 10.1016/j.bbr.2004.06.019 15617782

[B25] Follett PL Deng W Dai W Talos DM Massillon LJ Rosenberg PA Volpe JJ Jensen FE Glutamate receptor-mediated oligodendrocyte toxicity in periventricular leukomalacia: a protective role for topiramate J Neurosci 2004 24 4412 4420 10.1523/JNEUROSCI.0477-04.2004 15128855 PMC6729451

[B26] Scott RJ Hegyi L Cell death in perinatal hypoxic-ischaemic brain injury Neuropathol Appl Neurobiol 1997 23 307 314 10.1111/j.1365-2990.1997.tb01300.x 9292869

[B27] Chu K Lee ST Sinn DI Ko SY Kim EH Kim JM Kim SJ Park DK Jung KH Song EC Lee SK Kim M Roh JK Pharmacological induction of ischemic tolerance by glutamate transporter-1 (EAAT2) upregulation Stroke 2007 38 177 182 17122424 10.1161/01.STR.0000252091.36912.65

[B28] Johnston MV Trescher WH Ishida A Nakajima W Neurobiology of hypoxic-ischemic injury in the developing brain Pediatr Res 2001 49 735 741 10.1203/00006450-200106000-00003 11385130

[B29] Micu I Jiang Q Coderre E Ridsdale A Zhang L Woulfe J Yin X Trapp BD McRory JE Rehak R Zamponi GW Wang W Stys PK NMDA receptors mediate calcium accumulation in myelin during chemical ischaemia Nature 2006 439 988 992 16372019 10.1038/nature04474

[B30] Yuan X Eisen AM McBain CJ Gallo V A role for glutamate and its receptors in the regulation of oligodendrocyte development in cerebellar tissue slices Development 1998 25 2901 2914 10.1242/dev.125.15.2901 9655812

[B31] Follett PL Rosenberg PA Volpe JJ Jensen FE NBQX attenuates excitotoxic injury in developing white matter J Neurosci 2000 20 9235 9241 11125001 10.1523/JNEUROSCI.20-24-09235.2000 PMC6773002

[B32] Manning SM Talos DM Zhou C Selip DB Park HK Park CJ Volpe JJ Jensen FE NMDA receptor blockade with memantine attenuates white matter injury in a rat model of periventricular leukomalacia J Neurosci 2008 28 6670 6678 10.1523/JNEUROSCI.1702-08.2008 18579741 PMC2800040

[B33] Andine P Lehmann A Ellren K Wennberg E Kjellmer I Nielsen T Hagberg H The excitatory amino acid antagonist kynurenic acid administered after hypoxic-ischemia in neonatal rats offers neuroprotection Neurosci Lett 1988 90 208 212 10.1016/0304-3940(88)90813-0 3412643

[B34] Ford LM Sanberg PR Norman AB Fogelson MH MK-801 prevents hippocampal neurodegeneration in neonatal hypoxic-ischemic rats Arch Neurol 1989 46 1090 1096 2552968 10.1001/archneur.1989.00520460072016

[B35] Hagberg H Gilland E Diemer NH Andine P Hypoxia-ischemia in the neonatal rat brain: histopathology after post-treatment with NMDA and non-NMDA receptor antagonists Biol Neonate 1994 66 205 213 10.1159/000244109 7865635

[B36] McDonald JW Silverstein FS Johnston MV MK-801 protects the neonatal brain from hypoxic-ischemic damage Eur J Pharmacol 1987 140 359 361 10.1016/0014-2999(87)90295-0 2820765

[B37] Bar-Peled O Ben-Hur H Biegon A Groner Y Dewhurst S Furuta A Rothstein JD Distribution of glutamate transporter subtypes during human brain development J Neurochem 1997 69 2571 2580 9375691 10.1046/j.1471-4159.1997.69062571.x

[B38] Desilva TM Billiards SS Borenstein NS Trachtenberg FL Volpe JJ Kinney HC Rosenberg PA Glutamate transporter EAAT2 expression is up-regulated in reactive astrocytes in human periventricular leukomalacia J Comp Neurol 2008 508 238 248 10.1002/cne.21667 18314905 PMC2911955

[B39] Desilva TM Kinney HC Borenstein NS Trachtenberg FL Irwin N Volpe JJ Rosenberg PA The glutamate transporter EAAT2 is transiently expressed in developing human cerebral white matter J Comp Neurol 2007 501 879 890 10.1002/cne.21289 17311320 PMC8603423

[B40] Furuta A Rothstein JD Martin LJ Glutamate transporter protein subtypes are expressed differentially during rat CNS development J Neurosci 1997 17 8363 8375 9334410 10.1523/JNEUROSCI.17-21-08363.1997 PMC6573756

[B41] Kugler P Schleyer V Developmental expression of glutamate transporters and glutamate dehydrogenase in astrocytes of the postnatal rat hippocampus Hippocampus 2004 14 975 985 10.1002/hipo.20015 15390174

[B42] Fukamachi S Furuta A Ikeda T Ikenoue T Kaneoka T Rothstein JD Iwaki T Altered expressions of glutamate transporter subtypes in rat model of neonatal cerebral hypoxia-ischemia Brain Res Dev Brain Res 2001 132 131 139 11744117 10.1016/s0165-3806(01)00303-0

[B43] Dallas M Boycott HE Atkinson L Miller A Boyle JP Pearson HA Peers C Hypoxia suppresses glutamate transport in astrocytes J Neurosci 2007 27 3946 3955 10.1523/JNEUROSCI.5030-06.2007 17428968 PMC6672540

[B44] Nicholls D Attwell D The release and uptake of excitatory amino acids Trends Pharmacol Sci 1990 11 462 468 10.1016/0165-6147(90)90129-V 1980041

[B45] Ouyang YB Voloboueva LA Xu LJ Giffard RG Selective dysfunction of hippocampal CA1 astrocytes contributes to delayed neuronal damage after transient forebrain ischemia J Neurosci 2007 27 4253 4260 10.1523/JNEUROSCI.0211-07.2007 17442809 PMC3140959

[B46] Melzer N Meuth SG Torres-Salazar D Bittner S Zozulya AL Weidenfeller C Kotsiari A Stangel M Fahlke C Wiendl H A beta-lactam antibiotic dampens excitotoxic inflammatory CNS damage in a mouse model of multiple sclerosis PLoS One 2008 3 e3149 3160 10.1371/journal.pone.0003149 18773080 PMC2522272

[B47] Danbolt NC Glutamate uptake Prog Neurobiol 2001 65 1 105 10.1016/S0301-0082(00)00067-8 11369436

[B48] Chen W Aoki C Mahadomrongkul V Gruber CE Wang GJ Blitzblau R Irwin N Rosenberg PA Expression of a variant form of the glutamate transporter GLT1 in neuronal cultures and in neurons and astrocytes in the rat brain J Neurosci 2002 22 2142 2152 11896154 10.1523/JNEUROSCI.22-06-02142.2002 PMC2849837

[B49] Furness DN Dehnes Y Akhtar AQ Rossi DJ Hamann M Grutle NJ Gundersen V Holmseth S Lehre KP Ullensvang K Wojewodzic M Zhou Y Attwell D Danbolt NC A quantitative assessment of glutamate uptake into hippocampal synaptic terminals and astrocytes: new insights into a neuronal role for excitatory amino acid transporter 2 (EAAT2) Neuroscience 2008 157 80 94 10.1016/j.neuroscience.2008.08.043 18805467 PMC2775085

[B50] Holmseth S Scott HA Real K Lehre KP Leergaard TB Bjaalie JG Danbolt NC The concentrations and distributions of three C-terminal variants of the GLT1 (EAAT2; slc1a2) glutamate transporter protein in rat brain tissue suggest differential regulation Neuroscience 2009 162 1055 1071 10.1016/j.neuroscience.2009.03.048 19328838

[B51] Jansen EM Low WC Long-term effects of neonatal ischemic-hypoxic brain injury on sensorimotor and locomotor tasks in rats Behav Brain Res 1996 78 189 194 10.1016/0166-4328(95)00248-0 8864051

[B52] Duff P Antibiotic selection in obstetric patients Infect Dis Clin North Am 1997 11 1 12 10.1016/S0891-5520(05)70338-X 9067781

[B53] Bourget P Quinquis V Fernandez H Frydman R Clinical pharmacokinetics of ceftriaxone during the third trimester of pregnancy and study of its transplacental passage in two patients Pathol Biol (Paris) 1993 41 242 248 8332394

